# Periostin in Bronchiolitis Obliterans Syndrome after Lung Transplant

**DOI:** 10.3390/ijms251910423

**Published:** 2024-09-27

**Authors:** Hye Ju Yeo, Junho Kang, Yun Hak Kim, Woo Hyun Cho

**Affiliations:** 1 Division of Allergy, Pulmonary and Critical Care Medicine, Department of Internal Medicine, Transplant Research Center, Research Institute for Convergence of Biomedical Science and Technology, Pusan National University Yangsan Hospital, Yangsan 50612, Republic of Korea; hjyeo@pusan.ac.kr; 2 Department of Internal Medicine, School of Medicine, Pusan National University, Busan 43241, Republic of Korea; 3 Department of research, Keimyung University Donsan Medical Center, Daegu 42601, Republic of Korea; rkdwnsgh2002@nate.com; 4 Department of Anatomy, School of Medicine, Pusan National University, Yangsan 50612, Republic of Korea; yunhak10510@pusan.ac.kr; 5 Department of Biomedical Informatics, School of Medicine, Pusan National University, Yangsan 50612, Republic of Korea

**Keywords:** lung transplant, bronchiolitis obliterans syndrome, chronic lung allograft dysfunction, periostin, lung function

## Abstract

The utility of measuring serum periostin levels for predicting the occurrence of bronchiolitis obliterans syndrome (BOS) after lung transplantation remains underexplored. We analyzed differentially expressed genes (DEGs) between initially transplanted lung tissue and lung tissue with BOS from four patients. Periostin levels were assessed in 97 patients who had undergone lung transplantation 1 year post-transplantation and at the onset of BOS. The association between periostin levels and BOS, as well as their correlation with the decline in forced expiratory volume in one second (FEV1), was evaluated. Periostin levels in the BOS group were significantly higher than those in the control group (*p* < 0.001) and the stable group (*p* < 0.001). Periostin levels at the onset of BOS were significantly higher than those 1 year post-transplantation in the BOS group (*p* < 0.001). The serum periostin levels at the time of BOS diagnosis showed a positive correlation with the reduction in FEV1 (%) (r = 0.745, *p* < 0.001). The increase in the serum periostin levels at the time of BOS diagnosis compared with those 1 year post-transplantation was positively correlated with reduction in FEV1 (%) (r = 0.753, *p* < 0.001). Thus, serum periostin levels may serve as biomarkers for predicting a decline in lung function in patients with BOS after lung transplantation.

## 1. Introduction

Lung transplantation is a critical treatment option for end-stage lung disease; however, predicting long-term survival post-transplantation is challenging [1]. The reduction in long-term survival may be attributed to the development of bronchiolitis obliterans syndrome (BOS), which results in a progressive decline in lung function [2]. At present, BOS is diagnosed based on worsening dyspnea and spirometry findings [3]. Notably, functional decline is often detected after substantial damage has occurred to the lung tissue. Spirometry does not provide specific information regarding the pathology underlying this dysfunction; therefore, bronchoscopy and transbronchial biopsy must be performed to confirm the diagnosis of BOS. However, these multimodal diagnostic approaches have exhibited some limitations in asymptomatic patients and those with early-stage BOS. Thus, the identification of reliable blood-based biomarkers for facilitating the early detection of BOS is a critical area of research. 

The precise pathogenesis of BOS remains unclear; however, previous studies have suggested that epithelial–mesenchymal transition (EMT) may be a major mechanism underlying the occurrence of BOS [4,5]. EMT, defined as a process wherein epithelial cells are transformed into mesenchymal cells, is an important mechanism inducing tissue fibrosis in various pathological conditions [6,7]. Therefore, the genes related to BOS, especially those related to EMT and TGF-β signaling, were selected via differentially expressed gene (DEG) analysis. Among these, periostin was selected as the primary candidate gene.

Periostin, an extracellular matrix protein, plays a significant role in inducing inflammation and tissue remodeling processes [8] via its involvement in cell adhesion, migration, survival, and extracellular matrix remodeling. Periostin levels have shown associations with various inflammatory diseases and tissue fibrosis. Elevated periostin levels have been observed in patients with chronic airway diseases following fibrotic remodeling, including those with asthma and chronic obstructive pulmonary disease (COPD) [9,10,11]. Periostin levels are also elevated in patients with fibrotic organ diseases such as cardiac, liver, and renal fibrosis [12,13,14]. The expression of periostin, which is mainly controlled by fibroblasts, results in the excessive deposition of extracellular matrix proteins, such as collagen, during fibrosis [15]. Thus, periostin could serve as a potential biomarker for the early detection and fibrotic potential of BOS after lung transplantation owing to these properties. This study aimed to analyze the relationship between periostin levels in the blood and the occurrence of BOS after lung transplantation and evaluate the clinical utility of periostin as a biomarker for BOS.

## 2. Results

### 2.1. Gene Expression Profiling and Identification of Differentially Expressed Genes in BOS

**Figure 1:**
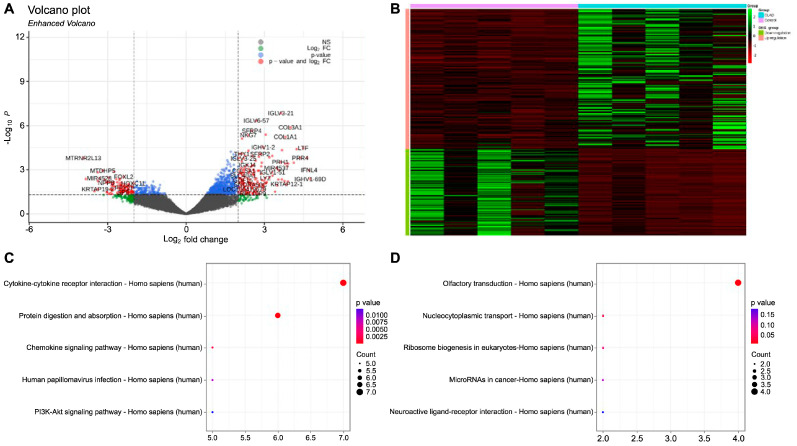
Comprehensive analysis of differential gene expression. (**A**): Volcano plot illustrating the differential expression analysis of 25,724 genes. The x-axis represents the log2 fold change, reflecting the magnitude of expression differences. The y-axis represents the negative logarithm (base 10) of the *p*-value, reflecting the statistical significance of the differential expression of each gene. The genes surpassing the log2 fold change threshold of 2 and *p*-value threshold of 0.05 are indicated in red (significantly differentially expressed), those only surpassing the *p*-value threshold are indicated in blue, those only surpassing the fold change threshold are indicated in green, and non-significant genes are indicated in black. (**B**): Heatmap of the expression patterns of the 332 differentially expressed genes across multiple samples. The x-axis categorizes individual samples, whereas the y-axis lists genes. The expression levels are color-coded, with upregulated genes indicated in varying shades of green and downregulated genes indicated in shades of red, facilitating a clear visual distinction between gene expression trends across different conditions. The color legend on the right side clarifies the gradation of the expression levels. (**C**): Pathway enrichment analysis of the upregulated genes in the KEGG database. The x-axis quantifies the number of genes involved in each pathway, whereas the y-axis identifies the pathways involved. The circle sizes represent the gene count in each pathway, and the color gradient indicates the significance of pathway enrichment, with darker shades denoting lower *p*-values. (**D**): Similar to Panel C, this plot shows the results of the pathway enrichment analysis for the downregulated genes using the same visual and analytical metrics.

**Figure 2:**
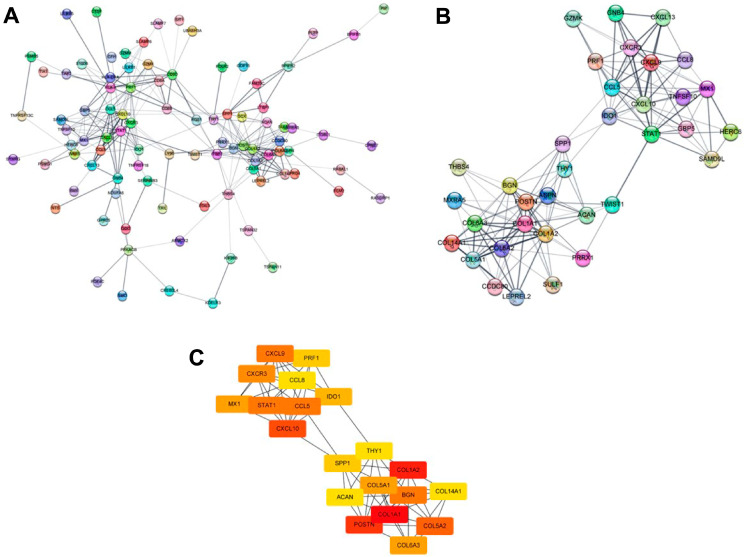
Network analysis of differentially expressed genes between lung tissue at the time of transplantation and BOS lung tissue. (**A**): Visualization of the protein–protein interaction (PPI) network constructed using the STRING database for 332 differentially expressed genes (DEGs). The network comprises 141 genes that exhibit known interactions, depicted as nodes connected by 359 linkages, reflecting the strength of the interactions and biological relationships among these genes. (**B**): Detailed representation of a highly connected module within the PPI network, featuring genes with the highest confidence interaction score of ≥9. This module was identified as central to BOS pathogenesis, with nodes corresponding to the genes shown in Figure 1B, underscoring their significant roles. (**C**): Analysis of hub genes within the PPI network comprising 20 genes that exhibit a high degree of connectivity, reflecting their pivotal role in gene interactions. Connectivity strength is represented by a color gradient from yellow to red, with red indicating the highest connection strength. These genes were selected based on their degree of connectivity, which ranges from 10 to 20, highlighting their critical influence within the network.

Gene expression profiling and differential expression analysis were performed in a dataset comprising RNA-Seq data of four terminally paired human lung samples obtained from patients with BOS. Supplementary Table S1 presents detailed demographic and clinical characteristics of the patients. The analysis led to identification of 332 DEGs, comprising 230 upregulated and 102 downregulated genes (Figure 1A). A heat map was used to visually represent the expression patterns of these DEGs (Figure 1B). Functional enrichment analysis was performed following the identification of DEGs to elucidate the biological roles of these genes. The upregulated DEGs were significantly enriched in biological terms, such as cytokine–cytokine receptor interaction (*p* = 0.0025), protein digestion and absorption (*p* = 0.0025), and the chemokine signaling pathway (*p* = 0.005) (Figure 1C). In contrast, the downregulated DEGs were significantly enriched in pathways, including olfactory transduction (*p* = 0.01), nucleocytoplasmic transport (*p* = 0.014), and ribosome biogenesis in eukaryotes (*p* = 0.024) (Figure 1D). A protein–protein interaction (PPI) network was constructed based on the previously identified interactions among the 230 upregulated DEGs to further explore the potential regulatory mechanisms of the identified DEGs. The network comprised 141 nodes and 359 edges, depicting the complex interactions among these proteins (Figure 2A). A highly interconnected module was identified within this network, comprising 35 nodes with a confidence score of ≥9 (Figure 2B). Twenty hub genes were identified using this module based on their degree of connectivity (ranging from 10 to 20), emphasizing their central roles in the network(Figure 2C). Lastly, 15 signature genes associated with the EMT and TGF-β signaling pathways were selected for further analysis. *Periostin*, which exhibited the highest hub gene score (degree = 17), was selected as the marker for subsequent validation.

### 2.2. Clinical Characteristics of the Validation Cohorts

The average age of participants in the control group was 61.7 ± 3.1 years, and 68.2% (n = 15) of the participants were men with no known comorbidities. A total of 131 patients underwent lung transplantation during the study period, and 29 died within 1 year (Supplementary Figure S1). Table 1 presents the baseline characteristics of the patients who had undergone lung transplantation. The average age of these patients was 56 ± 9.5 years, and 67% of these participants were men. Notably, 96.9% of the patients underwent bilateral lung transplantation. Idiopathic pulmonary fibrosis (IPF), accounting for 46.4% of cases, was the most common primary disease. All patients were receiving three immunosuppressive medications: tacrolimus, steroids, and mycophenolate. Twenty-five patients developed BOS during the follow-up period (median 1617 [1142–2029] days), whereas 72 patients remained stable. As of 20 June 2024, 40% and 16.7% of patients in the BOS and stable groups, respectively, had died. The median time to BOS was 919 [553.5–1287.5] days in the BOS group, whereas the median survival time was 2084 [1074.5–2824] days.

### 2.3. Serum Concentrations of TGF-Beta and Periostin

**Figure 3:**
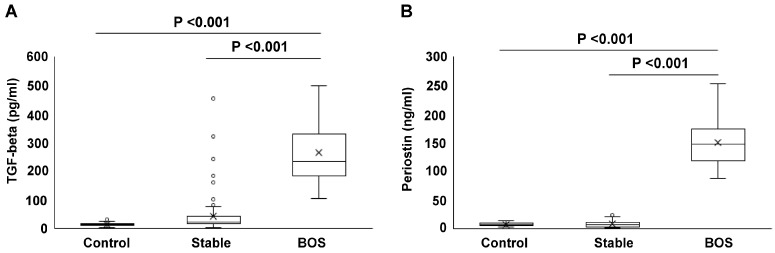
TGF-beta and periostin levels in the three groups. TGF-beta (**A**) and periostin (**B**) levels were measured in serum samples from healthy controls (n = 22) and an independent lung transplant cohort (n = 97). The lung transplant cohort was further divided into patients with bronchiolitis obliterans syndrome (BOS) (n = 25) and stable patients (n = 72). In the stable group, the serum levels of periostin and TGF-beta were measured one year after transplantation. In the BOS group, measurements were taken at the time of BOS diagnosis.

**Figure 4:**
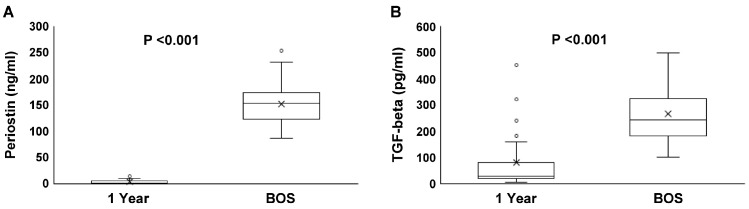
Periostin and TGF-beta between 1 year after lung transplantation and the time of BOS diagnosis in the BOS group. Periostin (**A**) and TGF-beta (**B**) were measured in the BOS group at both one year after transplantation and at the time of BOS diagnosis. Paired *t*-test results show that the periostin levels were significantly higher after the onset of BOS compared with that 1 year post-transplantation (3.7 vs. 153.0, *p* < 0.001). The TGF-β levels were also significantly higher after the onset of BOS compared with that 1 year post-transplantation (79.8 vs. 267.2, *p* < 0.001).

Serum TGF-beta levels in the BOS group were significantly higher than those in the control (267.2 ± 100.0 vs. 13.0 ± 7.2 pg/mL, *p* < 0.001) and stable (267.2 ± 100.0 vs. 25.9 ± 18.2 pg/mL, *p* < 0.001) groups (Figure 3A). Similarly, the periostin levels in the BOS group were higher than those in the control (153.0 ± 42.6 vs. 6.7 ± 2.7 ng/mL, *p* < 0.001) and stable (153.0 ± 42.6 vs. 8.8 ± 5.7 ng/mL, *p* < 0.001) groups (Figure 3B). The periostin levels in the BOS group at the time of BOS diagnosis were significantly higher than those 1 year post-transplantation (153.0 ± 42.6 vs. 3.7 ± 0.9 ng/mL, *p* < 0.001, Figure 4A). The TGF-beta levels at the time of BOS diagnosis were significantly higher than those 1 year post-transplantation (267.2 ± 100 vs. 79.8 ± 111.6 pg/mL, *p* < 0.001, Figure 4B).

### 2.4. Correlation between Serum Periostin Levels and Reduction in Lung Function

**Figure 5:**
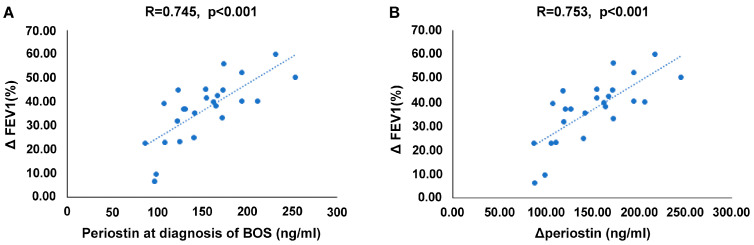
Correlation between lung function decline and periostin in the BOS group. (**A**). Correlation between the reduction rates of FEV1 (%) and periostin level at the time of BOS diagnosis. (**B**). Correlation between the reduction rates of FEV1 (%) and ΔPOSTN. Δ FEV1 (%) = (FEV1 at 1 year−FEV1 at BOS)/FEV1 at 1 year, BOS: bronchiolis obliterans syndrome. ΔPOSTN: ΔPOSTN = periostin at BOS−periostin at 1 year.

The analysis of the correlations between the serum periostin levels and the rate of FEV1 reduction at the time of BOS diagnosis revealed that the serum periostin levels at the time of BOS diagnosis were positively correlated with the reduction in FEV1 (%) (r = 0.745, *p* < 0.001) (Figure 5A). Furthermore, the increase in the serum periostin levels at the time of BOS diagnosis compared with that 1 year post-transplantation was positively correlated with the reduction rates of FEV1 (%) (r = 0.753, *p <* 0.001) (Figure 5B).

### 2.5. Serum Periostin Levels as a Biomarker for BOS after Lung Transplantation

**Figure 6:**
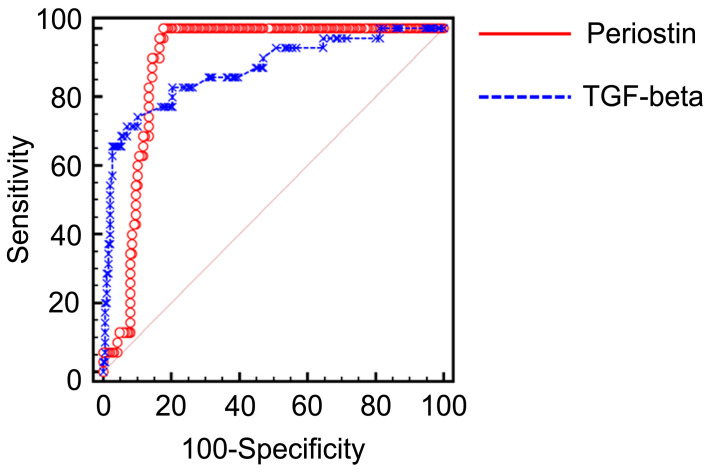
Serum periostin levels are a useful biomarker for predicting BOS after lung transplantation.

The association between the serum periostin levels and the occurrence of BOS was assessed by constructing receiver operating characteristic (ROC) curves (Figure 6) to determine whether the serum periostin levels reflected BOS after lung transplantation. The ROC curve analysis performed for the serum periostin levels (ng/mL) for predicting the possible presence of BOS yielded an area under the curve (AUC) of 0.899 (95% CI 0.859–0.939, *p* < 0.001). The best cut-off point for periostin for predicting the occurrence of BOS was 66.2 ng/mL, with a sensitivity of 100% and specificity of 82.35%. The ROC curve analysis for the serum TGF-beta levels (pg/mL) for predicting the possible presence of BOS yielded an AUC of 0.879 (95% CI 0.809–0.949, *p* < 0.001).

An ROC curve analysis was performed for the serum periostin levels (ng/mL) to predict the possible occurrence of BOS. The AUC of periostin was 0.899 (95% CI 0.859–0.939, *p* < 0.001). The AUC of TGF-beta was 0.879 (95% CI 0.809–0.949, *p* < 0.001). No significant differences were observed between the ROC curves of periostin and TGF-beta (*p* = 0.6278).

## 3. Discussion

The findings of this study indicate that, compared with those in normal individuals and stable recipients of lung transplantation, blood periostin levels are significantly elevated in patients who develop BOS following lung transplantation. Furthermore, the periostin levels increased from 1 year after lung transplantation to the onset of BOS, indicating a correlation with decline in lung function. Reduction in FEV1 from 1 year post-transplantation to the time of BOS diagnosis showed a significant association with the periostin levels and the change in periostin (Δperiostin) at the time of BOS diagnosis. These findings suggest that periostin is a valuable biomarker for the diagnosis and prediction of BOS progression.

The occurrence of BOS following lung transplantation remains a significant challenge. Current diagnostic methods, including the assessment of clinical symptoms, pulmonary function tests, and transbronchial lung biopsy, have exhibited limited utility in terms of early detection [3]. Therefore, there is an urgent requirement to develop blood biomarkers for predicting the occurrence of BOS to improve the management of patients undergoing lung transplantation. This study aimed to identify promising biomarker candidates for BOS via DEG analysis of the lung tissue at two different time points. The exact mechanism underlying BOS remains unknown; however, previous studies have suggested that EMT plays a critical role in the pathogenesis of BOS [4,5,16]. EMT, a process wherein epithelial cells transform into fibroblast-like cells, leads to structural changes in the airway and a long-term decline in lung function. EMT also induces the production of fibrosis-related proteins and accelerates lung fibrosis [17]. Thus, the genes involved in EMT and TGF-beta signaling were selected to validate the utility of periostin obtained from the blood samples of independent patients.

Periostin, an extracellular matrix protein, plays a critical role in the pathogenesis of BOS [18]. The present study also revealed that periostin is an important gene related to TGF-β and EMT in BOS. The expression of periostin can be upregulated by several cytokines, including TGF-β, which promotes the migration and activation of inflammatory cells in patients who have undergone transplantation [19,20]. However, the causal relationship between TGF-β and periostin in the development of BOS has not been fully elucidated. A persistent inflammatory environment is required to facilitate the onset and progression of BOS [2,21,22]. TGF-β, as an inflammatory cytokine, drives EMT and promotes the formation of fibrotic tissue [23,24]. Periostin is involved in cell–cell and cell–ECM interactions. Furthermore, it enhances myofibroblast activity, which is central to the fibrotic process and structural changes in the lung tissue affected by BOS [25]. Thus, periostin, along with TGF-β, may contribute to functional decline in lung tissue [26,27,28].

Previous in vitro studies have shown that periostin is basally secreted by airway epithelial cells or lung fibroblasts in response to IL-13 or IL-4, influencing epithelial cell function, epithelial–mesenchymal interactions, and ECM organization [29,30]. When primary human bronchial epithelial cells were either stimulated with periostin or overexpressed it, periostin was found to activate the TGF-β signaling pathway through a mechanism involving matrix metalloproteinases (MMPs) 2 and 9. Additionally, conditioned media from epithelial cells overexpressing periostin triggered TGF-β-dependent secretion of type 1 collagen by airway fibroblasts. Furthermore, mixing recombinant periostin with type 1 collagen solutions led to a significant increase in the elastic modulus of the collagen gel, indicating that periostin affects collagen fibrillogenesis or cross-linking, resulting in matrix stiffening. These findings underscore the important roles of epithelial-cell-derived periostin in TGF-β activation and collagen matrix elasticity, which are relevant to the pathophysiology of airway disease. To further elucidate the role of periostin in BOS, future in vitro experiments could focus on several key aspects. First, co-culture systems involving both airway epithelial cells and fibroblasts could be used to investigate the dynamic interactions between periostin and TGF-β in a controlled environment. Additionally, gene knockdown or CRISPR-Cas9 techniques could be employed to specifically inhibit periostin or TGF-β expression, allowing for a more detailed understanding of their respective contributions to fibrosis. Further studies could also examine the effects of periostin inhibitors on collagen cross-linking and matrix stiffening, which could offer potential therapeutic targets for preventing or mitigating BOS progression.

No previous study has specifically evaluated the role of periostin as a biomarker in patients who have undergone lung transplantation despite these insights. The TGF-β and periostin levels in patients with BOS were elevated compared with those in healthy controls and stable recipients of lung transplantation in the present study. These levels increased after the occurrence of BOS compared with that 1 year post-transplantation in patients with BOS. Notably, periostin levels showed a significant correlation with TGF-β levels (Supplementary Figure S2). Periostin and TGF-β effectively distinguished BOS from other conditions; however, no significant differences were observed between the two in terms of distinguishing BOS (AUC 0.899 and AUC 0.879, respectively; Figure 6). A decline in lung function showed a significant correlation with increased periostin levels. These findings indicate that periostin could serve as a valuable diagnostic biomarker for BOS. Early identification of patients at risk of developing BOS could facilitate the timely evaluation of potentially reversible causes, mitigate disease progression, and improve the long-term outcomes. Furthermore, predicting the occurrence of BOS based on periostin levels could aid in tailoring personalized immunosuppressive strategies to prevent the onset of BOS. Currently, there is no definitive treatment for BOS; however, early interventions such as immunosuppressive modulation, azithromycin therapy, and montelukast have shown potential benefits [31,32]. Measuring periostin levels could enable clinicians to identify patients at higher risk of developing BOS at an early stage, allowing for proactive therapeutic adjustments. Furthermore, implementing early monitoring protocols, such as additional lung function tests or imaging based on periostin levels, can help reduce unnecessary testing while detecting subtle changes in lung function before serious damage occurs. These proactive strategies may delay the onset of irreversible lung damage, prolong graft survival, improve patient outcomes, and significantly enhance the patient’s quality of life. 

This study had some limitations. The small sample size and observational study design limited the generalizability of the findings and their ability to establish causality. Further multicenter, large-scale studies must be conducted in the future to clarify the role of periostin and determine its clinical application as a biomarker. Nevertheless, the serum periostin levels showed a potential relationship with TGF-β, suggesting its involvement in fibrotic processes. Periostin exhibited great value as a biomarker in the present study, as it was derived via a DEG analysis using genetically identical tissues from two different time points: naïve donor lungs and those that had developed BOS. Furthermore, static measurements and dynamic changes in the periostin levels indicated its effectiveness as a useful biomarker for the early detection and prediction of BOS. Further understanding of the role of periostin in the fibrotic mechanism of BOS may help target its activity, mitigate fibrotic processes, and potentially slow or prevent the progression of BOS. Therefore, additional studies must be conducted in the future to identify the causal links and molecular pathways underlying periostin expression and the development of BOS after lung transplantation.

## 4. Materials and Methods

### 4.1. Patient Recruitment and Sample Collection for Differentially Expressed Gene Analysis

The DEGs were analyzed in four adult patients who had undergone lung re-transplantation between January 2019 and June 2020. RNA sequencing was performed using two samples: the naïve lung tissue sample at the time of transplantation and the lung tissue following the development of BOS. The DEGs between the lung tissues at transplantation and BOS lung tissues were analyzed. All tissue samples were obtained from the Pusan National University Yangsan Hospital (PNUYH) Biobank. All the tissue samples were anonymized, and the requirement for informed consent was waived. BOS was diagnosed according to the ISHLT guidelines [3].

### 4.2. RNA Sequencing Library Construction and Sequencing

DNA extraction was performed using the Maxwell^®^ 16 Formalin-Fixed, Paraffin-Embedded (FFPE) Plus LEV DNA Purification Kit (Promega Corp., Madison, WI, USA) at a final volume of 300 µL, in accordance with the manufacturer’s instructions. Supplementary Methods S1 provides details regarding DNA extraction, library construction, and sequencing. TRIzol, SDS, and the RNeasy Mini Kit (Qiagen, Hilden, Germany) were used to isolate and purify the RNA, with minor modifications, to extract RNA from the FFPE tissues in parallel. The protocol for obtaining DNase-free RNA included deparaffinization, proteinase K digestion, extraction, elution, or hydration procedures, and DNase treatment. The Agilent SureSelectXT RNA Direct Kit (Agilent Technologies, Santa Clara, CA, USA) was used in accordance with the manufacturer’s protocol to construct the sequencing libraries using 100 ng of RNA. Supplementary Methods S2 provides the details of RNA extraction, library construction, and sequencing. 

### 4.3. RNA-Seq Data Preprocessing and Analysis

The raw sequencing reads obtained via the RNA-Seq experiments were initially subjected to quality assessment using FastQC to determine the data integrity and quality metrics. Adapter sequences and low-quality bases were trimmed using Cutadapt (version 1.15), ensuring a minimum Phred score of 30 for the retained bases. This step played a critical role in removing potential sequencing artifacts that could affect subsequent analyses. The leaned reads were aligned with the human reference genome (GRCh38) using HISAT2 (version 2.2.1). The featureCount function of the subread package was used to quantify gene expression. Read counts were assigned to the genomic features by the software program based on the overlap between reads and annotated genes, thereby facilitating the accurate quantification of gene expression levels. The DESeq2 package was used to perform normalization and differential expression analyses of the quantified data. The genes were considered differentially expressed if they met the criteria of |log_2_ fold change| >2 and an adjusted *p*-value of <0.05, after adjusting for multiple testing performed using the Benjamini–Hochberg procedure. This stringent threshold ensured the identification of genes with significant changes in expression between conditions. A PPI network was constructed using data from the STRING database for the identified DEGs. Only interactions with a high confidence score (≥0.7) were included to ensure the reliability of the network. Gene Ontology (GO) and Kyoto Encyclopedia of Genes and Genomes (KEGG) pathway enrichment analyses were performed using the clusterProfiler package to explore the biological functions and pathways associated with the DEGs. This analysis identified overrepresented biological terms and pathways among the DEGs with a significance threshold set at *p* < 0.05.

### 4.4. Study Participants and Controls for Validation

Periostin was identified as a significant gene related to EMT and the TGF-beta pathway among the 20 hub DEGs. The periostin levels were evaluated in the blood of an independent validation cohort (n = 97) and healthy controls (n = 22). The validation cohort comprised patients who had undergone lung transplantation between December 2015 and December 2021. The serum samples were obtained from the PNUYH Biobank. Data regarding the demographic characteristics, age at the time of transplantation, type of transplantation, and underlying pathologies necessitating transplantation were extracted for all patients. The blood serum samples and results of the lung function tests 1 year post-transplantation were also collected for all patients. Furthermore, the lung function tests and collection of blood samples were performed at the time of diagnosing BOS as defined by the ISHLT guidelines [3]. Patients with concurrent infections at the time of serum sampling or with evidence of acute rejection were excluded from this study. This study was approved by the Institutional Review Board (IRB) of the Pusan National University Yangsan Hospital (approval number: 55-2024-002). The requirement for obtaining informed consent was waived owing to the retrospective nature of this study and the use of anonymized samples. 

### 4.5. Periostin and TGF-Beta Detection

Commercially available enzyme-linked immunosorbent assay (ELISA) kits were used in accordance with the manufacturer’s instructions (Human Periostin ELISA Kit (OSF2), Abcam, Cambridge, UK, AB213816; MyBioSource Inc., San Diego, CA, USA, MBS266143) to determine the serum concentrations of periostin and TGF-β. The concentrations of periostin and TGF-β were measured at 450 nm using an BioTek Epoch microplate spectrophotometer (Agilent Technologies, Santa Clara, CA, USA) and expressed in ng/mL and pg/mL, respectively.

### 4.6. Statistical Analysis

The continuous variables are presented as the means and standard deviations (M ± SD) or medians and quartiles (25th and 75th percentiles). The normality of the distribution of the variables was assessed using the Shapiro–Wilk test. The Chi-squared test was used to assess categorical variables. The periostin levels at 1 year and BOS status in patients with BOS were assessed using a paired *t*-test. The correlations between the periostin levels at the time of BOS diagnosis and the rates of decline in FEV1 (%) from 1 year post-lung transplantation to the time of BOS diagnosis were analyzed. In addition, the correlation between the increase in the periostin levels (ng/mL) and the rate of decline in the FEV1 (%) from 1 year post-lung transplantation to the time of BOS diagnosis was also analyzed. The best cut-off values for sensitivity and specificity were determined using a ROC curve analysis with the AUC. The best cut-off values for diagnosis were established using the Youden index [J = max (sensitivity + specificity − 1)]. All statistical analyses were performed using SPSS version 27.0 (IBM Corporation, Armonk, NY, USA) and R version 3.6.3 (R Foundation for Statistical Computing, Vienna, Austria). A *p*-value of <0.05 was considered statistically significant. 

## Figures and Tables

**Table 1:** Demographic and clinical data of the patients’ variables.

	Total (n = 97)
Age	56.0 ± 9.5
Male	65 (67)
BMI, kg/m^2^	21.0 ± 3.8
Type of lung transplantation	
Bilateral	94 (96.9)
Primary disease	
COPD	7 (7.2)
IPF	45 (46.4)
PPH	1 (1)
Non-IPF IIP	21 (21.6)
Bronchiectasis	2 (2.1)
Bronchiolitis Obliterans	13 (13.4)
Other	8 (8.2)
Pre-existing DSA	4 (4.1)
Previous acute rejection, A3	18 (18.6)
Donor age	40.3 ± 12.3
Donor smoking dose	6.6 ± 10.0
Donor PaO_2_/FiO_2_ ratio	443.2 ± 98.3

BMI, body mass index; COPD, chronic obstructive pulmonary disease; IPF, idiopathic pulmonary fibrosis; PPH, primary pulmonary hypertension; non-IPF, non-idiopathic pulmonary fibrosis; IIP, idiopathic interstitial pneumonia; DSA, donor-specific antibody. Data are presented as mean ± SD or n (%).

## Data Availability

All data generated or analyzed during this study are included in this published article and its files in Supplementary Materials.

## Supplementary Materials

The following supporting information can be downloaded at https://www.mdpi.com/article/10.3390/ijms251910423/s1.

## References

[1] Kulkarni H.S., Cherikh W.S., Chambers D.C., Garcia V.C., Hachem R.R., Kreisel D., Puri V., Kozower B.D., Byers D.E., Witt C.A. (2019). Bronchiolitis obliterans syndrome-free survival after lung transplantation: An International Society for Heart and Lung Transplantation Thoracic Transplant Registry analysis. J. Heart. Lung. Transpl..

[2] Yu Y., Kim Y.H., Cho W.H., Kim D., So M.W., Son B.S., Yeo H.J. (2024). Unique Changes in the Lung Microbiome following the Development of Chronic Lung Allograft Dysfunction. Microorganisms.

[3] Verleden G.M., Glanville A.R., Lease E.D., Fisher A.J., Calabrese F., Corris P.A., Ensor C.R., Gottlieb J., Hachem R.R., Lama V. (2019). Chronic lung allograft dysfunction: Definition, diagnostic criteria, and approaches to treatment-A consensus report from the Pulmonary Council of the ISHLT. J. Heart. Lung. Transpl..

[4] Rahman M., Ravichandran R., Bansal S., Sanborn K., Bowen S., Eschbacher J., Sureshbabu A., Fleming T., Bharat A., Walia R. (2022). Novel role for tumor suppressor gene, liver kinase B1, in epithelial-mesenchymal transition leading to chronic lung allograft dysfunction. Am. J. Transpl..

[5] Hodge S., Holmes M., Banerjee B., Musk M., Kicic A., Waterer G., Reynolds P.N., Hodge G., Chambers D.C. (2009). Posttrans-plant bronchiolitis obliterans syndrome is associated with bronchial epithelial to mesenchymal transition. Am. J. Transpl..

[6] Rout-Pitt N., Farrow N., Parsons D., Donnelley M. (2018). Epithelial mesenchymal transition (EMT): A universal process in lung diseases with implications for cystic fibrosis pathophysiology. Respir. Res..

[7] Willis B.C., duBois R.M., Borok Z. (2006). Epithelial origin of myofibroblasts during fibrosis in the lung. Proc. Am. Thorac. Soc..

[8] Conway S.J., Izuhara K., Kudo Y., Litvin J., Markwald R., Ouyang G., Arron J.R., Holweg C.T., Kudo A. (2014). The role of periostin in tissue remodeling across health and disease. Cell. Mol. Life. Sci..

[9] Krasilnikova S.V., Tush E.V., Frolov P.A., Ovsyannikov D.Y., Terentyeva A.B., Kubysheva N.I., Eliseeva T.I. (2021). Periostin as a Biomarker of Allergic Inflammation in Atopic Bronchial Asthma and Allergic Rhinitis (a Pilot Study). Sovrem. Tekhnologii. Med..

[10] Nejman-Gryz P., Górska K., Paplińska-Goryca M., Proboszcz M., Krenke R. (2020). Periostin and Thymic Stromal Lymphopoietin-Potential Crosstalk in Obstructive Airway Diseases. J. Clin. Med..

[11] Nanishi M., Fujiogi M., Freishtat R.J., Hoptay C.E., Bauer C.S., Stevenson M.D., Camargo C.A., Hasegawa K. (2022). Serum periostin among infants with severe bronchiolitis and risk of developing asthma: A prospective multicenter cohort study. Allergy.

[12] Hwang J.H., Yang S.H., Kim Y.C., Kim J.H., An J.N., Moon K.C., Oh Y.K., Park J.Y., Kim D.K., Kim Y.S. (2017). Experimental Inhibition of Periostin Attenuates Kidney Fibrosis. Am. J. Nephrol..

[13] Kumar P., Smith T., Raeman R., Chopyk D.M., Brink H., Liu Y., Sulchek T., Anania F.A. (2018). Periostin promotes liver fi-brogenesis by activating lysyl oxidase in hepatic stellate cells. J. Biol. Chem..

[14] Gil H., Goldshtein M., Etzion S., Elyagon S., Hadad U., Etzion Y., Cohen S. (2022). Defining the timeline of periostin up-regulation in cardiac fibrosis following acute myocardial infarction in mice. Sci. Rep..

[15] Crawford J., Nygard K., Gan B.S., O'Gorman D.B. (2015). Periostin induces fibroblast proliferation and myofibroblast persistence in hypertrophic scarring. Exp. Dermatol..

[16] Renaud-Picard B., Vallière K., Toussaint J., Kreutter G., El-Habhab A., Kassem M., El-Ghazouani F., Olland A., Hirschi S., Porzio M. (2020). Epithelial-mesenchymal transition and membrane microparticles: Potential implications for bronchiolitis obliterans syndrome after lung transplantation. Transpl. Immunol..

[17] Salton F., Volpe M.C., Confalonieri M. (2019). Epithelial-Mesenchymal Transition in the Pathogenesis of Idiopathic Pulmonary Fibrosis. Medicina.

[18] Müller C., Rosmark O., Åhrman E., Brunnström H., Wassilew K., Nybom A., Michaliková B., Larsson H., Eriksson L.T., Schultz H.H. (2021). Protein Signatures of Remodeled Airways in Transplanted Lungs with Bronchiolitis Obliterans Syndrome Obtained Using Laser-Capture Microdissection. Am. J. Pathol..

[19] Masuoka M., Shiraishi H., Ohta S., Suzuki S., Arima K., Aoki S., Toda S., Inagaki N., Kurihara Y., Hayashida S. (2012). Periostin promotes chronic allergic inflammation in response to Th2 cytokines. J. Clin. Investig..

[20] Kavvadas P., Dussaule J.C., Chatziantoniou C. (2014). Searching novel diagnostic markers and targets for therapy of CKD. Kidney. Int. Suppl..

[21] Greenland N.Y., Deiter F., Calabrese D.R., Hays S.R., Kukreja J., Leard L.E., Kolaitis N.A., Golden J.A., Singer J.P., Greenland J.R. (2022). Inflammation on bronchoalveolar lavage cytology is associated with decreased chronic lung allograft dysfunction-free survival. Clin. Transpl..

[22] Dugger D.T., Fung M., Hays S.R., Singer J.P., Kleinhenz M.E., Leard L.E., Golden J.A., Shah R.J., Lee J.S., Deiter F. (2021). Chronic lung allograft dysfunction small airways reveal a lymphocytic inflammation gene signature. Am. J. Transpl..

[23] Willis B.C., Borok Z. (2007). TGF-beta-induced EMT: Mechanisms and implications for fibrotic lung disease. Am. J. Physiol. Lung. Cell. Mol. Physiol..

[24] Lee J.H., Massagué J. (2022). TGF-β in developmental and fibrogenic EMTs. Semin. Cancer. Biol..

[25] Ackerman J.E., Adjei-Sowah E., Korcari A., Muscat S.N., Nichols A.E.C., Buckley M.R., Loiselle A.E. (2023). Identification of Periostin as a critical niche for myofibroblast dynamics and fibrosis during tendon healing. bioRxiv.

[26] Ashley S.L., Wilke C.A., Kim K.K., Moore B.B. (2017). Periostin regulates fibrocyte function to promote myofibroblast differentiation and lung fibrosis. Mucosal. Immunol..

[27] Nanri Y., Nunomura S., Terasaki Y., Yoshihara T., Hirano Y., Yokosaki Y., Yamaguchi Y., Feghali-Bostwick C., Ajito K., Murakami S. (2020). Cross-Talk between Transforming Growth Factor-β and Periostin Can Be Targeted for Pulmonary Fibrosis. Am. J. Respir. Cell. Mol. Biol..

[28] Tirunavalli S.K., Kuncha M., Sistla R., Andugulapati S.B. (2023). Targeting TGF-β/periostin signaling by sesamol ameliorates pulmonary fibrosis and improves lung function and survival. J. Nutr. Biochem..

[29] Sidhu S.S., Yuan S., Innes A.L., Kerr S., Woodruff P.G., Hou L., Muller S.J., Fahy J.V. (2010). Roles of epithelial cell-derived periostin in TGF-beta activation, collagen production, and collagen gel elasticity in asthma. Proc. Natl. Acad. Sci. USA..

[30] Takayama G., Arima K., Kanaji T., Toda S., Tanaka H., Shoji S., McKenzie A.N., Nagai H., Hotokebuchi T., Izuhara K. (2006). Periostin: A novel component of subepithelial fibrosis of bronchial asthma downstream of IL-4 and IL-13 signals. J. Allergy. Clin. Immunol..

[31] Williams K.M., Pavletic S.Z., Lee S.J., Martin P.J., Farthing D.E., Hakim F.T., Rose J., Manning-Geist B.L., Gea-Banacloche J.C., Comis L.E. (2022). Prospective Phase II Trial of Montelukast to Treat Bronchiolitis Obliterans Syndrome after Hematopoietic Cell Transplantation and Investigation into Bronchiolitis Obliterans Syndrome Pathogenesis. Transpl. Cell. Ther..

[32] Hao X., Peng C., Lian W., Liu H., Fu G. (2022). Effect of azithromycin on bronchiolitis obliterans syndrome in posttransplant recipients: A systematic review and meta-analysis. Medicine.

