# Supplementary material for: Periostin in Bronchiolitis Obliterans Syndrome after Lung Transplant
Source: Int J Mol Sci. 2024 Sep 27;25(19):10423. doi: 10.3390/ijms251910423 (PMC11477235; doi:10.3390/ijms251910423)
Supplement: Supplementary file 1 [file ijms-25-10423-s001.zip › ijms-3193469-supplementary.pdf]

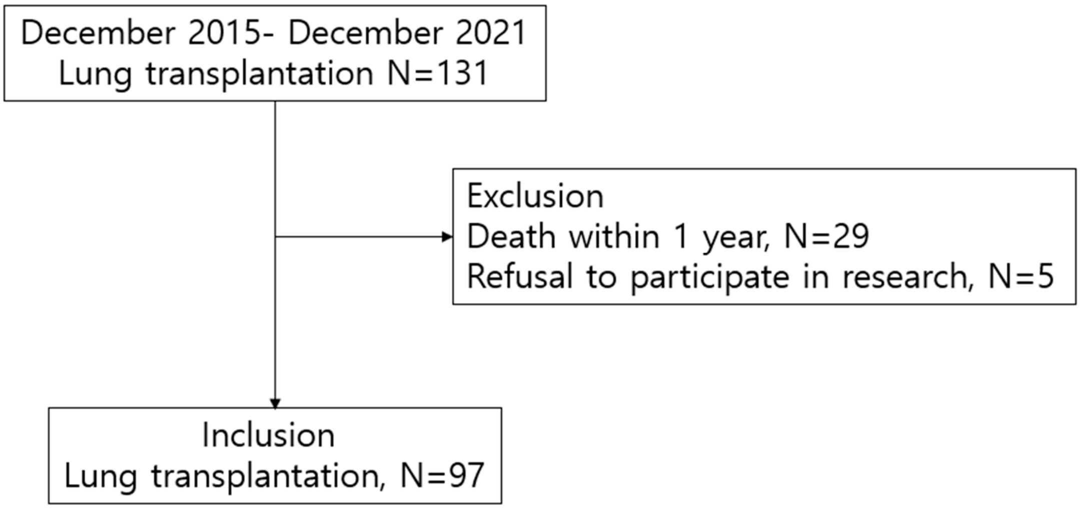

Supplementary Figure S1. Patient flow

A total of 97 lung transplant patients were included in the blood-based validation study.

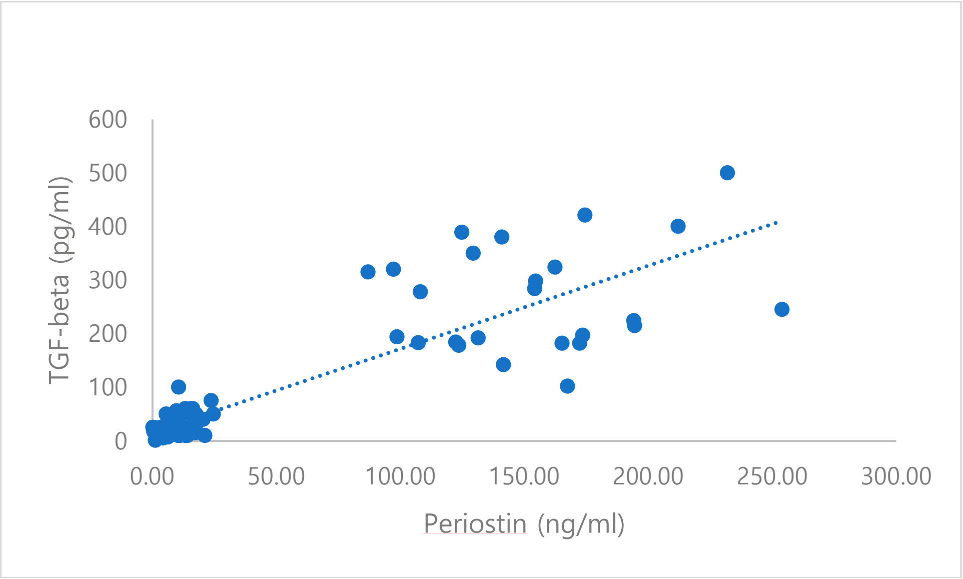

Supplementary Figure S2. Relation between TGF- beta and periostin

A significant correlation was confirmed between tgf beta levels and periostin levels ($r=0.879$, $p<0.001$).

Supplementary Method S1. DNA Extraction, Library Construction, and Sequencing

The integrity of the genomic DNA was assessed using agarose gel electrophoresis. Genomic DNA (gDNA) was quantified using the Quant-IT PicoGreen assay (Invitrogen, Thermo Fisher Scientific, Waltham, MA, USA). Sequencing libraries were prepared using a TruSeq DNA Nano Library Prep Kit (Illumina, Inc., San Diego, CA, USA) in accordance with the manufacturer's instructions. gDNA (100 ng) was fragmented using adaptive-focused acoustic technology (Covaris LLC, Woburn, MA, USA). The fragmented DNA was subjected to end-repair, resulting in the formation of 5'-phosphorylated, blunt-ended, double-stranded DNA molecules. The size of the DNA fragments was selected using a bead-based technique. The resulting fragments underwent the addition of a single 'A' base and ligation of TruSeq indexing adapters. The purified libraries were quantified using quantitative polymerase chain reaction (qPCR) following the qPCR Quantification Protocol Guide (KAPA Library Quantification Kits for Illumina Sequencing Platforms). The libraries were further qualified using an Agilent Technologies 2200 TapeStation (Agilent Technologies, Santa Clara, CA, USA). Paired-end sequencing with a read length of 2 × 150 bp was performed using the Macrogen (Seoul, Korea) and NovaSeq platforms (Illumina, Inc.).

Supplementary Method S2. RNA extraction, library construction, and sequencing

Total RNA was extracted from cell samples using the TRIzol reagent kit (Invitrogen) in accordance with the manufacturer's protocol. The integrity of the RNA samples was assessed using the TapeStation RNA ScreenTape (Agilent, Wilmington, DE, USA). The RNA libraries were independently prepared using the Illumina TruSeq Stranded Total RNA Library Prep Gold Kit (Illumina Inc., San Diego, CA, USA). The cleaved RNA fragments were copied into first-strand cDNA using SuperScript II reverse transcriptase (Invitrogen, Carlsbad, CA, USA) with random primers. The qualified libraries were sequenced on an Illumina NovaSeq platform (Illumina Inc., San Diego, CA, USA). The total RNA concentration was calculated using the Quant-IT RiboGreen Assay Kit (Invitrogen, Waltham, MA, USA). The samples were run on a TapeStation RNA ScreenTape to determine the DV200 values (percentage of RNA fragments >200 bp).

A total of 100 ng of total RNA was used for sequencing library construction using the Agilent SureSelect RNA Direct Kit (Agilent, Wilmington, DE, USA) in accordance with the manufacturer's protocol. Total RNA was fragmented into small pieces using divalent cations at elevated temperatures. The cleaved RNA fragments were copied into first-strand cDNA using random primers. This step was followed by second-strand cDNA synthesis. The cDNA fragments were subjected to end repair, the addition of a single "A" base, and adapter ligation. The products were purified and enriched using PCR to create a cDNA library. The Agilent SureSelect XT Human All Exon v6+UTRs Kit was used in accordance with the standard Agilent SureSelect Target Enrichment protocol to capture the human exonic regions. A cDNA library (25 ng) was mixed with hybridization buffers, blocking mixes, RNase block, and 5 μL of SureSelect XT Human All Exon v6+UTRs capture library. Hybridization was performed at 65°C using the heated thermal cycler lid option at 105°C for 24 hours in a PCR machine to capture baits. The captured library was washed and subjected to a second round of PCR amplification. The final purified product was quantified via qPCR in accordance with the qPCR Quantification Protocol Guide (KAPA Library Quantification Kits for Illumina Sequencing platforms) and qualified using the TapeStation DNA ScreenTape D1000. The indexed libraries were sequenced using a NovaSeq system (Illumina, Inc.) with paired-end (2 × 100 bp) sequencing performed by Macrogen, Inc. (Daejeon, Korea).

Supplementary Table S1. Characteristics of the patients included in the DEG analysis

Variables	Total (N=4)
Age, years	57 [40.3-66.3]
Male	2 (50)
BMI, kg/m^2	23.8 [19.8-25.9]
Comorbidities	
Diabetes	3 (75)
Hypertension	1 (25)
Chronic kidney disease	2 (50)
Indication for first lung transplant	
IPF	3 (75)
PPH	1 (25)
Induction- basiliximab	4 (100)
Maintenance -triple	4 (100)
Tacrolimus	4 (100)
Mycophenolate	3 (75)
Azathioprine	1 (25)
Primary graft dysfunction immediately after transplant	0
Acute rejection	0
BOS free survival, days	693.5 [328.5–853.8]

BMI, body mass index; IPF, idiopathic pulmonary fibrosis; PPH, primary pulmonary hypertension; BOS, bronchiolitis obliterans syndrome.

Data presented as median[interquartile range] or N(%).
